# Oxytocin ameliorates early life stress-induced anxiety-like behavior by normalizing corticotropin-releasing hormone neuron activity in the PVN

**DOI:** 10.1016/j.isci.2026.115349

**Published:** 2026-03-12

**Authors:** Cuihui Li, Yunlong Xu, Bingchun Lin, Xiaoyan Huang, Zhixi Huang, Fuxiang Zheng, Lin Zhu, Yingjie Zhu, Chuanzhong Yang

**Affiliations:** 1 Department of Neonatology, Shenzhen Maternity and Child Healthcare Hospital, Women and Children’s Medical Center, Shenzhen School of Clinical Medicine, Southern Medical University, Shenzhen, Guangdong Province, China; 2 Shenzhen Key Laboratory of Maternal and Child Health and Diseases, Shenzhen 518055, China; 3 Shenzhen Key Laboratory of Drug Addiction, Shenzhen Neher Neural Plasticity Laboratory, The Brain Cognition and Brain Disease Institute, Shenzhen Institutes of Advanced Technology, Chinese Academy of Sciences, Shenzhen 518055, China; 4 The fourth Clinical Medical College of Guangzhou University of Chinese Medicine, Shenzhen 510833, Guangdong, China

**Keywords:** biological sciences, behavioral neuroscience

## Abstract

Maternal separation (MS), a prevalent form of early life stress (ELS) exposure, elevates vulnerability to anxiety disorders in later developmental stages. Despite its clinical relevance, the neural mechanisms remain poorly defined. In this study, we demonstrate that ELS-exposed offspring exhibit sustained anxiety-like behaviors. Through integrated c-Fos immunostaining and whole-cell patch-clamp recordings, we identified hyperactivation of corticotropin-releasing hormone (CRH) neurons in the paraventricular nucleus (PVN) of the hypothalamus (PVN^CRH^) of ELS mice, characterized by enhanced neuronal activity and increased intrinsic excitability. Critically, chemogenetic suppression of PVN^CRH^ neurons effectively alleviated anxiety-like phenotypes in ELS mice. Systemic oxytocin (OXT) administration reversed ELS-associated anxiety behaviors and normalized both synaptic hyperexcitability and intrinsic hyperactivation of PVN^CRH^ neurons. Importantly, chemogenetic activation of PVN^CRH^ neurons abolished OXT’s anxiolytic effects. These findings delineate a neurobiological pathway through which ELS programs anxiety susceptibility, and identify OXT-mediated regulation of PVN^CRH^ neuronal excitability as a potential therapeutic strategy for stress-related psychopathologies.

## Highlights

• ELS induces anxiety-like behavior and enhances the excitability of PVN^CRH^ neurons
• Chemogenetic inhibition of PVN^CRH^ neurons alleviates ELS-induced anxiety
• Oxytocin reverses ELS-induced anxiety and intrinsic hyperexcitability in PVN^CRH^ neurons
• Chemogenetic activation of PVN^CRH^ neurons abolishes the anxiolytic effect of oxytocin

## Graphical abstract

**Figure:**
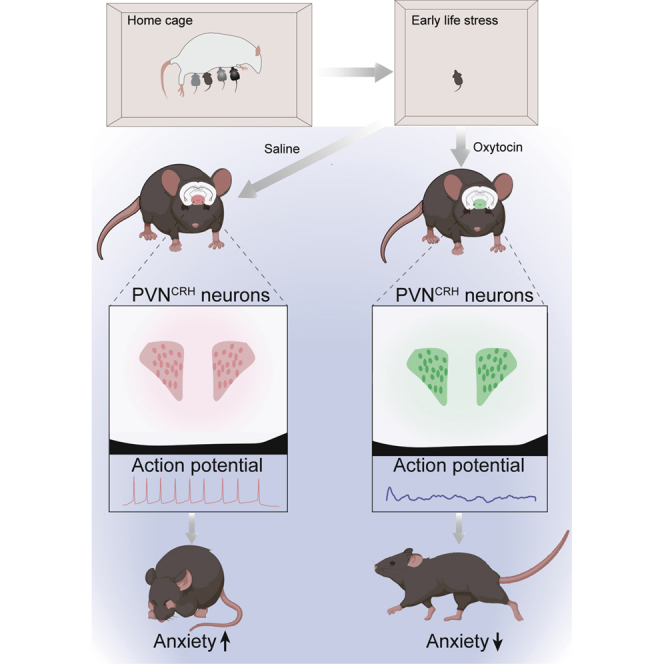

## Introduction

Early life exposure to chronic stressors induces persistent detrimental effects on neurodevelopmental processes and biological system networks.^1^^,^^2^ Early life stress (ELS) significantly elevates the lifetime risk of psychiatric disorders, representing a major etiological factor in psychopathology development.^1^ Numerous epidemiological studies demonstrate a robust association between ELS and enhanced vulnerability to subsequent psychopathology.^3^^,^^4^ Adverse life events elevate childhood/adolescent depression risk approximately 2.5-fold.^5^ Maternal separation (MS) constitutes a predominant ELS paradigm,^3^^,^^6^ serving as a well-established preclinical model for investigating behavioral disorders related to early stress.^7^^,^^8^ Anxiety, as core psychopathological components, are prevalent in ELS-exposed adolescent populations.^9^

Corticotrophin-releasing hormone (CRH) neurons in the paraventricular nucleus (PVN) of the hypothalamus (PVN) represent a primary neuronal ensemble coordinating stress-responsive neuroendocrine, autonomic, and behavioral processes. These neurons mediate hormonal regulation via activation of the hypothalamic-pituitary-adrenal (HPA) axis while directly modulating limbic circuitry and autonomic nuclei through efferent projections.^10^^,^^11^ Experimental results demonstrate that ELS induces persistent dysregulation of the HPA axis, abnormalities of CRH signaling, and impairment of glucocorticoid receptor-mediated feedback mechanisms.^12^ These perturbations significantly affect neurotransmitter homeostasis and induce neural dysfunction across multiple brain regions.^13^^,^^14^^,^^15^^,^^16^ However, it has remained unclear how PVN^CRH^ neurons respond to ELS, and the role of these neurons in mediating ELS-induced anxiety remains unclear.

As a nonapeptide hormone, oxytocin (OXT) is mainly synthesized in the PVN and supraoptic nucleus (SON) of the hypothalamus. Human cohort studies reveal a strong negative correlation between anxiety levels and circulating OXT levels.^17^^,^^18^^,^^19^ Moreover, OXT knockout (Oxt-KO) mice display anxiety-like phenotypes.^20^ One study has confirmed the therapeutic efficacy of exogenous OXT in psychiatric disorders.^21^ Thus, these findings indicate that OXT may serve as a key stress-buffering regulator in anxiety homeostasis, representing a promising therapeutic target for anxiety disorders.^22^ However, it is unclear whether OXT affects ELS-induced anxiety, and the neurobiological mechanism linking MS-induced anxiety to OXT remains unclear.

## Results

### ELS increases the excitability of PVN^CRH^ neurons and promotes anxiety-like behavior

**Figure 1:**
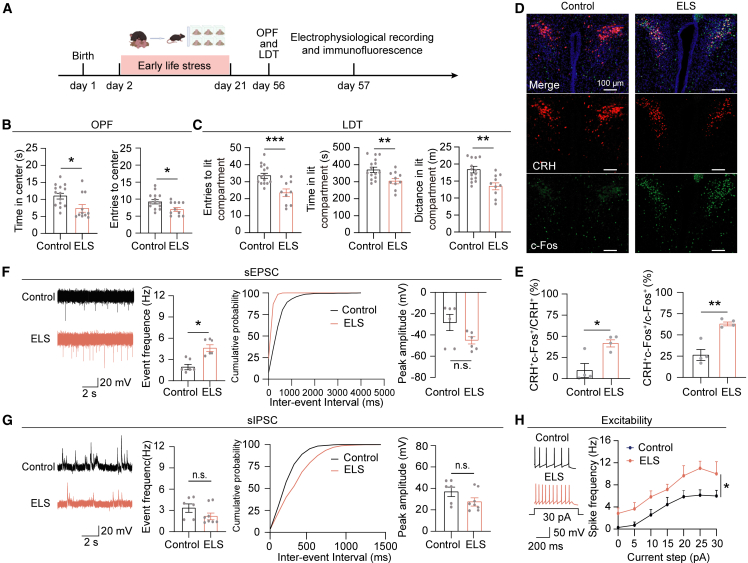
ELS increases the excitability of PVN^CRH^ neurons and promotes anxiety-like behavior (A) Schematic and timeline depicting the ELS paradigm and experiences. (B) Quantification of the duration in the center zone (left, *p* = 0.0177, unpaired *t* test,t = 2.555, df = 23) and the center area entries in the OFT (right, *p* = 0.0153, unpaired *t* test, t = 2.620, df = 23) of control (*n* = 14) and ELS groups (*n* = 11). (C) Quantification of the entries in the lit compartment (left, *p* = 0.0007, unpaired *t* test, t = 3.897, df = 23), the duration in the lit compartment (middle, *p* = 0.0092, unpaired *t* test, t = 2.843, df = 23) and the distance in the lit compartment in the LDT (right, *p* = 0.0026, unpaired *t* test, t = 3.373, df = 23) of control (*n* = 15) and ELS groups (*n* = 10). (D) Representative images illustrate co-labeling of c-Fos-positive neurons (green) and CRH-positive neurons (red) in the PVN following ELS. Scale bars, 100 μm. (E) Quantification of PVN^CRH^ neurons that were c-Fos-positive (left, *p* = 0.0139, unpaired *t* test, t = 3.437, df = 6) and c-Fos-positive neurons that were CRH-positive (right, *p* = 0.0017, unpaired *t* test, t = 5.380, df = 6) in control (*n* = 4) and ELS groups (*n* = 4). (F) Representative traces of sEPSCs in PVN^CRH^ neurons from control (top), ELS group (bottom) mice. The frequency of sEPSCs was increased in the ELS group, but not in the control group (*p* = 0.0022, Mann-Whitney test; control *n* = 6, ELS *n* = 6). Cumulative probability of sEPSCs inter-event intervals across the two groups. No difference in sEPSCs amplitude was observed in the two groups (*p* = 0.2403, Mann-Whitney test; control *n* = 6, ELS *n* = 6). (G) Representative traces of sIPSCs in PVN^CRH^ neurons from control (top), ELS (bottom) mice. No difference observed in the frequency of sIPSCs in the two groups (*p* = 0.1315, Mann-Whitney test; control *n* = 6, ELS *n* = 8). Cumulative probability distribution for inter-event intervals of sIPSCs. No difference observed in the amplitude of sIPSCs in the two groups (*p* = 0.1002, Mann-Whitney test; control *n* = 6, ELS *n* = 8). (H) Representative traces show the action potential firing of PVN^CRH^ neurons at 5 pA current injection from control (top), ELS (bottom) mice. Input-output curve reveals increased the intrinsic excitability of PVN^CRH^ neurons in the ELS mice [*F* (6, 66) = 0.3050, *p* = 0.9322, two-way repeated measures ANOVA followed by post hoc Holm-Sidak’s test, control *n* = 7, ELS *n* = 6]. Data are presented as mean ± SEM. ∗*p* < 0.05, ∗∗*p* < 0.01, and ∗∗∗*p* < 0.001; n.s., no significant difference.

To investigate the long-term behavioral consequences of ELS, C57BL/6 mice were exposed to ELS protocols at the age of postnatal days (PND) 2 (Figure 1A), followed by behavioral tests conducted. The ELS offspring mice model was established as follows: C57BL/6 mouse pups were subjected to ELS for 4 h daily during PND 2–5, followed by extended separation for 8 h daily from PND 6 to 16. The ELS offspring were weaned on PND 17. Control offspring maintained standard maternal rearing conditions until PND 21. Subsequently, we performed the open field test (OFT) and light/dark box test (LDT) to assess the impact of ELS on the anxiety of those mice. In the OFT, the ELS mice showed reduced entries into the center zone, and spent less time in the center zone compared to the control group (Figure 1B). Similarly, in the LDT, ELS mice displayed fewer entries into the lit compartment, spent less time in the lit compartment, and traveled shorter distances in the lit compartment compared to the control mice (Figure 1C). These results indicate that ELS mice exhibit increased anxiety.

In order to examine whether PVN^CRH^ neurons participate in the ELS-induced anxiety-like behaviors, we performed c-Fos staining to assess PVN neuron activity following ELS treatment (Figure 1A). ELS induced significant c-Fos expression in the PVN^CRH^ neurons, with 61% of c-Fos-positive neurons being CRH-positive (Figures 1D and 1E). Among all PVN^CRH^ neurons, about 38% were c-Fos-positive, suggesting the activation of PVN^CRH^ neurons by ELS (Figures 1D and 1E). To further examine the synaptic mechanisms underlying responses triggered by ELS, we used CRH:AI14 transgenic mice to label CRH-positive neurons with red fluorescent protein tdTomato. Whole-cell patch-clamp recordings were performed on acute hypothalamic slices to measure the spontaneous excitatory postsynaptic currents (sEPSCs) and spontaneous inhibitory postsynaptic currents (sIPSCs) in PVN^CRH^ neurons. We found that significant increases in the frequency of sEPSCs in the ELS group compared to the control group, while the amplitude was unaffected (Figure 1F). Both the frequency and amplitude of the sIPSCs in the ELS mice were not significantly different from those of control mice (Figure 1F). Together, these electrophysiological results suggest ELS-induced synaptic dyshomeostasis characterized by increased excitatory inputs. Intrinsic excitability of PVN^CRH^ neurons was quantified through depolarizing steady currents (0–30 pA, in 5 pA increments) (Figure 1H). The PVN^CRH^ neurons in the ELS mice exhibited significantly enhanced excitability compared to the control group (Figure 1H), as demonstrated by the elevated input-output curve. Overall, these results suggest that the excitatory synaptic inputs and intrinsic neuronal excitability of PVN^CRH^ neurons were potentiated in ELS mice.

### Chemogenetic inhibition of PVN^CRH^ neurons alleviates ELS-induced anxiety-like behaviors

**Figure 2:**
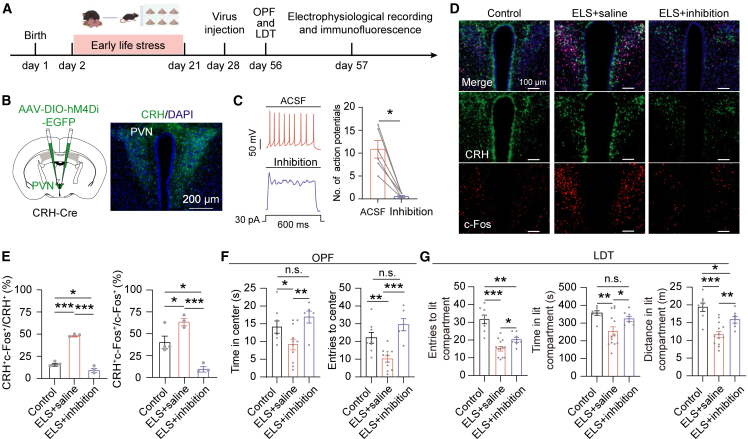
Chemogenetic inhibition of PVN^CRH^ neurons alleviates ELS-induced anxiety-like behavior (A) Schematic and timeline depict the ELS paradigm and experiences. (B) Schematic illustrating viral strategy for targeted inhibition of PVN^CRH^ neurons (left). Representative images depict the expression of hM4Di-EGFP in the PVN of CRH-Cre mice. Scale bars, 200 μm (right). (C) Representative traces displaying that CNO treatment blocked action potential firing in hM4Di-expressing PVN^CRH^ neurons (left). Quantification of action potentials evoked by a step current injection (30 pA) under artificial cerebrospinal fluid (ACSF) and CNO treatment [W = −21.00, *p* = 0.0313, Wilcoxon matched-pairs signed-rank test (two-tailed), *n* = 6]. (D) Representative images displaying c-Fos-positive neurons (green) in control, ELS+saline, and ELS+inhibition groups. Scale bars, 100 μm. (E) Percentage of hM4Di-expressing PVN^CRH^ neurons that were c-Fos-positive [*F* (2, 6) = 153.0, *p* < 0.0001, one-way ANOVA followed by post hoc Holm-Sidak’s test, control *n* = 4, ELS+saline *n* = 3, ELS+inhibition *n* = 3] and c-Fos-positive neurons that were CRH-positive [*F* (2, 7) = 19.43, *p* = 0.0014, one-way ANOVA followed by post hoc Holm-Sidak’s test] for control (*n* = 4), ELS+saline (*n* = 3), and ELS+inhibition (*n* = 3) groups. (F) Quantification of the duration in the center zone [left, *F*(2,21) = 5.492, *p* = 0.0121, one-way ANOVA followed by post hoc Holm-Sidak’s test] and the center area entries [right, *F* (2, 21) = 11.76, *p* = 0.0004, one-way ANOVA followed by post hoc Holm-Sidak’s test] for control (*n* = 7), ELS+saline (*n* = 11) and ELS+inhibition (*n* = 6) groups. (G) Quantification of the entries in the lit compartment [left, *F* (2, 25) = 24.89, *p* < 0.0001, one-way ANOVA followed by post hoc Holm-Sidak’s test], the duration in the lit compartment [middle, *F* (2, 25) = 5.766, *p* = 0.0087, one-way ANOVA followed by post hoc Holm-Sidak’s test] and the distance in the lit compartment [right, *F* (2, 25) = 16.08, *p* < 0.0001, one-way ANOVA followed by post hoc Holm-Sidak’s test] for control (*n* = 8), ELS+saline (*n* = 13) and ELS+inhibition (*n* = 7) groups. Data are presented as mean ± SEM. ∗*p* < 0.05, ∗∗*p* < 0.01, and ∗∗∗*p* < 0.001; n.s., no significant difference.

Given the increased activity and excitability of the PVN^CRH^ neurons in ELS mice, we hypothesized that the chemogenetic inhibition of this neuronal population might reverse ELS-induced anxiety. PVN^CRH^ neurons were transduced with inhibitory designer receptors exclusively activated by designer drugs (DREADDs) hM4Di after ELS exposure (Figures 2A and 2B). *Ex vivo* slices electrophysiology confirmed clozapine-N-oxide (CNO, 10 μM) effectively suppressed action potential firing of PVN^CRH^ neurons (Figure 2C), validating the inhibitory efficacy of CNO. CNO was administered 30 min before each behavioral test and c-Fos experiments, and an equal amount of saline was given to controls. Systemic CNO administration (5 mg/kg, i.p.) attenuated ELS-induced c-Fos activation in PVN^CRH^ neurons (Figures 2D and 2E). Moreover, ELS resulted in anxiety-like behavior, while the inhibition of PVN^CRH^ neurons reversed anxiety levels in ELS mice (Figures 2F and 2G). The results indicate that the inhibition of PVN^CRH^ neurons alleviates ELS-induced anxiety.

### OXT alleviates ELS-induced anxiety

**Figure 3:**
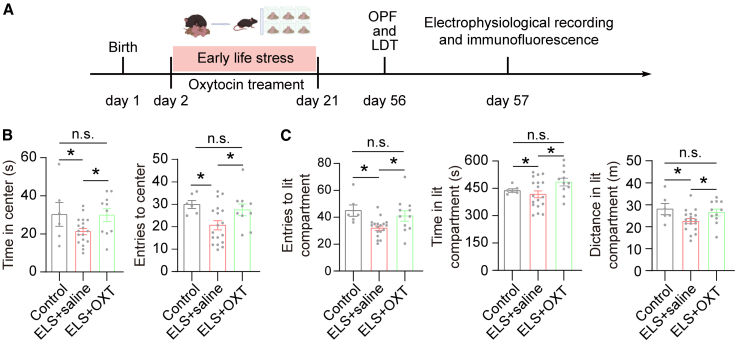
OXT alleviates ELS-induced anxiety (A) Schematic and timeline depict the ELS paradigm, OXT treatment, and experiences. (B) Quantification of the duration in the center zone [left, *F*(2,31) = 3.293, *p* = 0.0505, one-way ANOVA followed by post hoc Holm-Sidak’s test] and the center area entries [right, *F*(2, 31) = 4.217, *p* = 0.0240, one-way ANOVA followed by post hoc Holm-Sidak’s test] for control (*n* = 6), ELS+saline (*n* = 18), and ELS+OXT (*n* = 10) groups. (C) Quantification of the entries in the lit compartment [left, *F*(2, 32) = 5.501, *p* = 0.0088, one-way ANOVA followed by post hoc Holm-Sidak’s test], the duration in the lit compartment [middle, *F* (2, 27.48) = 4.318, *p* = 0.023, one-way ANOVA (Brown-Forsythe correction for unequal variances), Games-Howell post-hoc tests were used instead of Holm-Sidak due to heteroscedasticity] and the distance in the lit compartment [right, *F*(2, 32) = 3.549, *p* = 0.0405, one-way ANOVA followed by post hoc Holm-Sidak’s test] for control (*n* = 6), ELS+saline (*n* = 18) and ELS+OXT groups (*n* = 11). Data are presented as mean ± SEM. ∗*p* < 0.05; n.s., no significant difference.

To determine the effect of OXT on ELS-induced anxiety, the ELS mice (PND 2) received daily OXT administration (1 mg/kg, i.p.) until PND 21 prior to performing behavior assays, electrophysiological recording, and immunofluorescence (Figure 3A). Anxiety-related behaviors were quantified using OFT and LDT. Compared to ELS mice treated with saline, the ELS mice treated with OXT exhibited increased entries into the center zone and spent more time in the center zone compared to the ELS mice in the OPF (Figure 3B). In the LDT, the ELS mice treated with OXT displayed more entries into the lit compartment, spent more time in the lit compartment, and traveled longer distances in the lit compartment compared to the control group (Figure 3C). These results indicate that OXT alleviates the ELS-induced anxiety.

### OXT reverses ELS-induced neuronal activity and synaptic potential of PVN^CRH^ neurons

**Figure 4:**
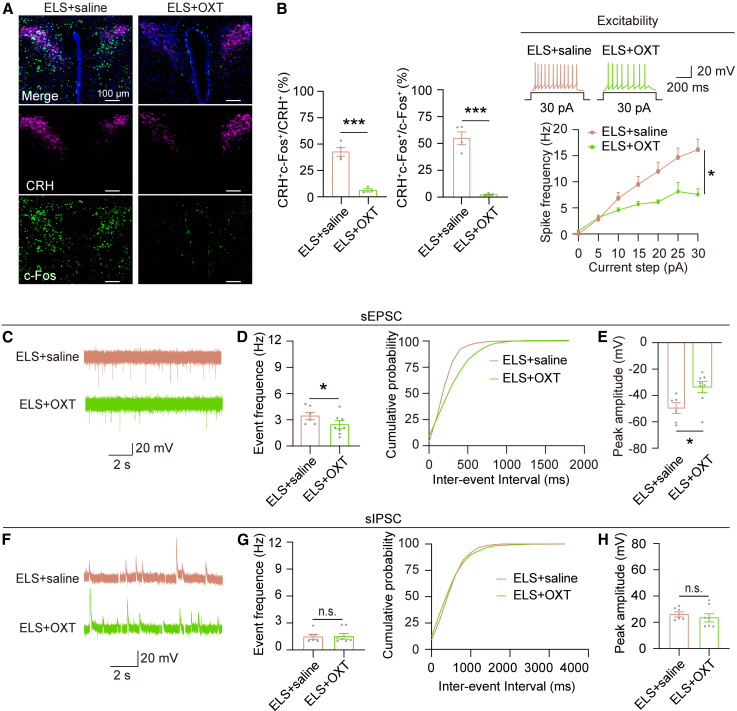
OXT reverses ELS-induced neuronal activity and synaptic potential of PVN^CRH^ neurons (A) Representative images illustrate co-labeling of c-Fos-positive neurons (green) and CRH-positive neurons (magenta) in the PVN following OXT treatment. Scale bars, 100 μm. (B) Quantification of PVN^CRH^ neurons that were c-Fos-positive (left, *p* = 0.0007, unpaired *t* test, t = 7.334, df = 5) and c-Fos-positive neurons that were CRH-positive (right, *p* = 0.0007, unpaired *t* test, t = 7.437, df = 5) in ELS+saline (*n* = 4) and ELS+OXT (*n* = 3) groups. (C–E) Representative traces of sEPSCs (C) in PVN^CRH^ neurons from the ELS+saline group (top) and ELS+OXT group (bottom). The frequency of sEPSCs (D) was decreased in the ELS+OXT group (*p* = 0.0743, Mann-Whitney test; ELS+saline *n* = 4, ELS+OXT *n* = 3). Cumulative probability distribution of sEPSCs inter-event intervals (D) across the two groups. The amplitude of sEPSCs (E) was decreased in the ELS+OXT group (*p* = 0.0050, Mann-Whitney test; ELS+saline *n* = 4, ELS+OXT *n* = 3). (F–H) Representative traces of sIPSCs (F) in PVN^CRH^ neurons from the ELS+saline group (top) and the ELS+OXT group (bottom). No difference observed in the frequency of sIPSCs (G) in the two groups (*p* = 0.8939, Mann-Whitney test; ELS+saline *n* = 6, ELS+OXT *n* = 7). Cumulative probability distribution for inter-event intervals of sIPSCs (G). No difference observed in the amplitude of sIPSCs (H) in the two groups (*p* = 0.6964, Mann-Whitney test; ELS+saline *n* = 6, ELS+OXT *n* = 7). (I) Representative traces showing the action potential firing of PVN^CRH^ neurons at 5 pA current injection from the ELS+saline group (top) and the ELS+OXT group (bottom). Input-output curve reveals decreased intrinsic excitability of PVN^CRH^ neurons in the ELS+OXT mice [*F* (6, 78) = 3.245, *p* = 0.0067, Two-way repeated measures ANOVA followed by post hoc Holm-Sidak’s test, ELS+saline *n* = 8, ELS+OXT *n* = 7]. Data are presented as mean ± SEM. ∗*p* < 0.05 and ∗∗∗*p* < 0.001; n.s., no significant difference.

ELS significantly increased c-Fos expression in the PVN^CRH^ neurons (Figures 4A and 4B), while OXT decreased the c-Fos expression (Figures 4A and 4B). To further examine the synaptic transmission in PVN^CRH^ neurons, we used CRH:AI14 mice for targeted patch clamp recording. We recorded the sEPSCs and sIPSCs in PVN^CRH^ neurons from MS mice treated with saline and MS mice treated with OXT. We found that both the frequency of sEPSCs was significantly decreased in the ELS mice treated with OXT compared to ELS mice treated with saline (Figures 4C and 4D), and the amplitude was also significantly decreased in the ELS mice treated with OXT (Figures 4C and 4E). However, both the frequency (Figures 4F and 4G) and amplitude (Figures 4F and 4H) of sIPSCs in the ELS mice treated with OXT were not different from those of ELS mice treated with saline. We also assessed the intrinsic excitability of PVN^CRH^ neurons by depolarizing steady currents (Figure 4I). The PVN^CRH^ neurons in the ELS mice treated with OXT exhibited reduced excitability compared to the ELS mice (Figure 4I). In conclusion, these results indicate OXT rescued ELS-induced the activity and excitability of PVN^CRH^ neuron.

### Inhibition of PVN^CRH^ neurons is required for the anxiolytic effects of OXT

**Figure 5:**
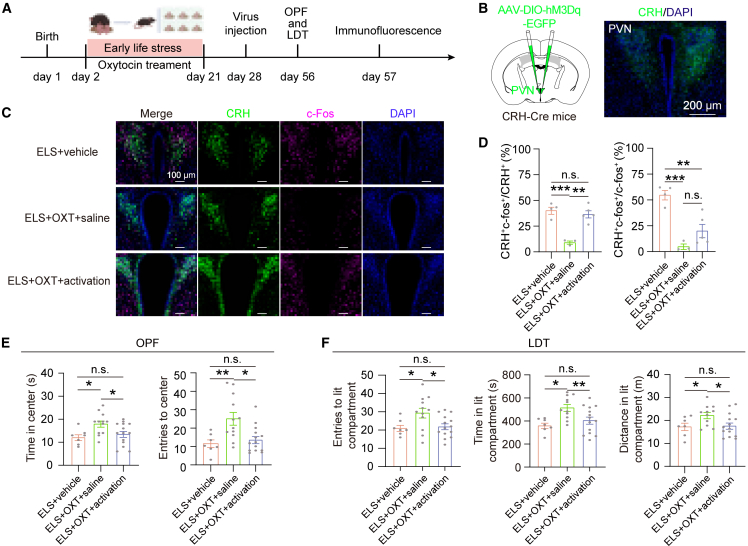
Inhibition of PVN^CRH^ neurons is required for the anxiolytic effects of OXT (A) Schematic and timeline depicting the ELS paradigm, OXT treatment, and experiences. (B) Schematic illustrating viral strategy for targeted activation of PVN^CRH^ neurons (left). Representative image depicts the expression of hM3Dq-EGFP in the PVN of CRH-Cre mice. Scale bars, 200 μm (right). (C) Representative images display c-Fos-positive neurons (green) in ELS+vehicle, ELS+OXT+saline, and ELS+OXT+ activation groups. Scale bars, 100 μm. (D) Percentage of hM3Dq-expressing PVN^CRH^ neurons that were c-Fos-positive [left, *F* (2, 9) = 22.50, *p* = 0.0003, one-way ANOVA followed by post hoc Holm-Sidak’s test] and c-Fos-positive neurons that were CRH-positive [right, *F* (2, 9) = 19.40, *p* = 0.0005, one-way ANOVA followed by post hoc Holm-Sidak’s test] for ELS+vehicle (*n* = 4), ELS+OXT+saline (*n* = 3), and ELS+OXT+ activation (*n* = 5) groups. (E) Quantification of the duration in the center zone [left, *F* (2, 30) = 4.649, *p* = 0.0174, one-way ANOVA followed by post hoc Holm-Sidak’s test] and the center area entries [right, *F* (2, 30) = 7.086, *p* = 0.0030, one-way ANOVA followed by post hoc Holm-Sidak’s test] for ELS+vehicle (*n* = 7), ELS+OXT+saline (*n* = 12), and ELS+OXT+ activation (*n* = 14) groups. (F) Quantification of the entries in the lit compartment [left, *F* (2, 30) = 4.499, *p* = 0.0195, one-way ANOVA followed by post hoc Holm-Sidak’s test], the duration in the lit compartment [middle, *F* (2, 30) = 4.499, *p* = 0.0195, one-way ANOVA followed by post hoc Holm-Sidak’s test] and the distance in the lit compartment [right, F (2, 29) = 3.468, *p* = 0.0446, one-way ANOVA followed by post hoc Holm-Sidak’s test] of ELS+vehicle (*n* = 7), ELS+OXT+saline (*n* = 12), and ELS+OXT+ activation (*n* = 14) groups. Data are presented as mean ± SEM. ∗*p* < 0.05 and ∗∗∗*p* < 0.001; n.s., no significant difference.

Given the decreased activity and excitability of the PVN^CRH^ neurons in ELS mice treated with OXT, we sought to examine the causal role of PVN^CRH^ neurons in mediating OXT’s anxiolytic effects. PVN^CRH^ neurons with the excitatory hM3Dq virus were transduced, followed by subjecting the mice to ELS and OXT treatment (Figures 5A and 5B). An i.p. injection of CNO (0.5 mg/kg) induced robust c-Fos activation in PVN^CRH^ neurons of ELS mice treated with OXT (Figures 5C and 5D). In the OFT, CNO treatment significantly reduced entries into the center zone, and decreased the time spent in the center zone in the ELS mice treated with OXT (Figure 5E). In the LDT, CNO treatment significantly reduced entries into the lit compartment, decreased the time spent in the lit compartment, and decreased traveled distances in the lit compartment in the ELS mice treated with OXT (Figure 5F). These data conclusively demonstrate that PVN^CRH^ neuronal hyperactivation abolishes OXT’s ameliorative effects on ELS-induced anxiety.

## Discussion

In summary, our findings demonstrate that ELS leads to anxiety-like behaviors and induces synaptic dyshomeostasis characterized by enhanced excitatory inputs in PVN^CRH^ neurons, together with increased intrinsic excitability. These electrophysiological alterations correlate with behavioral phenotypes of anxiety, consistent with clinical observations linking ELS to heightened psychopathology risk.^4^^,^^5^^,^^9^ Chemogenetic inhibition PVN^CRH^ neurons abolished ELS-induced anxiety phenotypes. Notably, the dual modulations of synaptic plasticity and intrinsic excitability suggest that ELS-induced anxiety arises from PVN^CRH^ neuron activity.

Several studies have demonstrated the activation of the HPA axis due to ELS. For example, Van Bodegom et al. highlighted that exposure to MS induces a hyper-reactive HPA axis, characterized by increased CRH and adrenocorticotropic hormone (ACTH) levels, which correlates with anxiety-like behaviors.^12^ Similarly, ELS can result in maladaptive HPA-axis functioning, leading to excessive stress responses and a heightened vulnerability to psychopathologies in child development and behavior.^23^ However, a number of studies have shown that moderate levels of early-life adversity can lead to enhanced resilience to stress in adulthood. This phenomenon is thought to involve adaptive changes in the stress response system, including alterations in the HPA axis, neuroplasticity, and neurochemical signaling that enhance coping mechanisms in response to later-life stressors.^24^ Our study builds on this body of work by focusing on the specific neuronal populations involved in mediating the anxiety-like behaviors associated with ELS. While previous studies have largely concentrated on the broad changes in HPA axis activity, we focus on the PVN^CRH^ neurons, which are central in HPA axis regulation, and investigate how synaptic plasticity and intrinsic excitability of these neurons are altered by ELS. This approach allows us to provide a mechanistic understanding of how ELS affects neuronal activity and leads to long-term behavioral consequences. In particular, our study provides more insights through electrophysiological techniques, offering a deeper, more mechanistic understanding of how MS affects neuronal activity at the level of synaptic transmission and intrinsic excitability in PVN^CRH^ neurons. This goes beyond the c-Fos expression results and provides a direct link between the neurobiological alterations in these neurons and the MS-induced anxiety. Our findings highlight that enhanced excitatory inputs and increased intrinsic excitability of PVN^CRH^ neurons are key drivers of anxiety-like behaviors induced by ELS.

The stressor used in the experiments is not only the MS period, but also the early weaning, which is performed at PND17, whereas controls are weaned at PD21. Early weaning might be an important factor, together with the MS period. Previous studies in rodents have shown that prolonged separation from the dam, combined with early weaning, leads to more reliable and consistent behavioral changes, such as increased anxiety-like behaviors and depressive symptoms, making the model suitable for studying the neurobiological mechanisms underlying these behaviors.^25^

The therapeutic efficacy of OXT in reversing ELS-induced neural and behavioral abnormalities provides compelling evidence for its neuromodulatory potential. Sarah J Baracz et al. demonstrated that OXT treatment in adolescent rats exposed to ELS reduces anxiety, methamphetamine intake, and stress-induced behavior, suggesting OXT’s therapeutic potential in addiction treatment.^26^ By restoring synaptic homeostasis through the suppression of excitatory transmission and intrinsic excitability, OXT effectively normalized PVN^CRH^ neuronal hyperactivity. The reverse of OXT’s anxiolytic effects upon the chemogenetic reactivation of PVN^CRH^ neurons underscores the centrality of this cell population in mediating therapeutic outcomes. However, we found that OXT specifically modulates excitatory transmission without affecting inhibitory inputs, pointing to circuit-specific mechanisms that warrant further investigation. Additionally, the findings resonate with clinical studies demonstrating impaired OXT signaling in anxiety patients. For example, studies have demonstrated reduced cerebrospinal fluid OXT levels exposed to maltreatment during childhood.^27^ Men with a history of ELS had abnormal cortisol response to exogenous OXT.^28^

OXT treatment during the entire ELS period (from PND2 to PND21) is a critical aspect in our study. By targeting the whole postnatal developmental period, we are influencing not only the acute stress response but also the developmental plasticity of key stress-regulatory circuits. OXT signaling in the hypothalamus can directly repress CRH transcription via OXTR and dampen PVN stress output,^29^ and OXTR expression is developmentally regulated, implying that neonatal OXT can have enduring organizational effects. For the OXT dose, basal circulating OXT of rodents is 400–600 pg/mL from PND7 to PND21.^30^ Our results indicate OXT treatment (1 mg/kg, i.p.) raises blood OXT level of mice to 1907 ng/mL, and it is therefore a supraphysiological dose.

This work identifies PVN^CRH^ neurons as a promising therapeutic node for ELS-related psychopathology. The reversibility of both synaptic and intrinsic abnormalities by OXT treatment supports the development of targeted neuromodulatory strategies for the treatment of ELS-induced anxiety. In addition to OXT treatment in ELS-induced anxiety, alternative approaches, such as circuit-specific manipulation, could be explored based on these mechanistic insights. Importantly, the temporal dissociation between OXT intervention and anxiety phenotypes suggests lasting modifications to anxiety. Future studies should focus on molecular mediators of OXT’s effects and the upstream circuit of PVN^CRH^ neurons. Additionally, individual differences in OXT receptor distribution and stress resilience mechanisms may explain variable treatment responses, highlighting the need for personalized therapeutic strategies in anxiety disorders. OXT may exert direct effects on CRH neurons in the PVN. OXT receptors (OXTRs) are expressed in PVN^CRH^ neurons, and activation of these receptors can modulate neuronal excitability and synaptic plasticity.^31^ Activation of OXTR has been shown to reduce excitability and CRH release in these neurons,^31^ which may help normalize the HPA axis dysfunction caused by early-life stress. This modulation occurs possibly via G protein-coupled signaling pathways, including the inhibition of cAMP production, which leads to a reduction in neuronal firing and stress hormone release.^32^

Our study offers more insights into the neurobiological mechanisms underlying anxiety-like behaviors induced by ELS: (1) Our study identifies PVN^CRH^ neurons as a central node in the regulation of anxiety-like behavior induced by ELS. (2) We showed that ELS-induced anxiety was associated with a dual modulation of synaptic plasticity and intrinsic excitability in PVN^CRH^ neurons, a previously underexplored mechanism that could provide an understanding of how ELS alters behavior. (3) We demonstrate that OXT can reverse both synaptic and intrinsic neuronal abnormalities in PVN^CRH^ neurons induced by ELS. (4) Neonatal oxytocin intervention and long-term anxiety-like behavior further support the lasting effects of early life interventions on stress regulation circuits.

### Limitations of the study

While the study identifies the therapeutic potential of OXT in ELS-related anxiety through regulating the activity and excitability of PVN^CRH^ neurons in individuals, several limitations will be considered. First, the longitudinal efficacy of OXT administration, particularly regarding therapeutic outcomes in ELS mice at post-8-week age, cannot be conclusively determined. Second, this investigation focused exclusively on male mice, precluding extrapolation to females. Sexual dimorphism in HPA axis regulation may critically influence findings. Future studies incorporating female mice are essential to delineate sex-specific regulatory mechanisms in ELS.

## Resource availability

### Lead contact

Further information and requests for resources and reagents should be directed to and will be fulfilled by the lead contact, Chuanzhong Yang (yangczgd@smu.edu.cn).

### Materials availability

This study did not generate new unique reagents.

### Data and code availability

• All data reported in this paper will be shared by the lead contact upon request.
• This paper does not report original code.
• Any additional information requested to re-analyze the data reported in this paper is available from the lead contact upon request.

## Acknowledgments

This study is supported by the Shenzhen Maternity and Child Healthcare Hospital Intramural Research Funding Program (FYA2022007), the 10.13039/501100012151Sanming Project of Medicine in Shenzhen (SZSM202211001), the Shenzhen Fund for Guangdong Provincial Highlevel Clinical Key Specialties (No. SZGSP009), the Shenzhen Medical Research Fund (A2503076), the Shenzhen Science and Technology Program (JCYJ20240813115000002, JCYJ20250604150000001), the Shenzhen Key Laboratory of Maternal and Child Health and Diseases (ZDSYS20230626091559006), the 10.13039/501100021171Guangdong Basic and Applied Basic Research Foundation (2023A1515110345), and the China Postdoctoral Science Foundation General Program (2023M733658).

## Author contributions

C.Y. and Y.X. conceived the study. C.L. performed virus injection experiments. B.L. and X.H. conducted genotyping and immunofluorescence experiments. C.L. and X.H. performed behavioral experiments. C.L. performed the DREADD manipulation experiments. C.L. performed early life stress experiments. Y.X. performed Electrophysiological experiments. Y.X., Y.Z., and C.Y. wrote the manuscript with input from all the authors.

## Declaration of interests

The authors declare no competing interests.

## STAR★Methods

### Key resources table

**Table:** 

REAGENT or RESOURCE	SOURCE	IDENTIFIER
**Antibodies**		

Chicken anti-c-Fos	Synaptic Systems	Cat226009; RRID: AB_2943525
Rabbit anti-CRH	Peninsula Laboratories	Cat#T-4037; RRID: AB_2314240
Rabbit anti-c-Fos	Cell Signaling Technology	Cat#2250; RRID: AB_2247211
Alexa Fluor Cy5 Goat anti-Rabbit	Invitrogen	Cat#A21070; RRID: AB_2535731
Alexa Fluor 647 Goat anti Chicken	Invitrogen	Cat#A21449; RRID: AB_2535866
Alexa Fluor 488 Goat anti-Chicken	Invitrogen	Cat#A11039; RRID: AB_2534096
Alexa Fluor 555 Goat anti-Rabbit	Invitrogen	Cat#A-21428; RRID: AB_2535849
Alexa Fluor 488 Goat anti-Rabbit	Jackson Immuno Research	Cat#111-547-003; RRID: AB_2338058

**Bacterial and virus strains**		

AAV2/9-hSyn-DIO-hM4D(Gi)-EGFP-WPRE-pA	Taitool Company	Cat#S0193-9
AAV2/9-hsyn-DIO-hM3D(Gq)-ER2-P2A-EGFP-WPRE-PA	Taitool Company	Cat#S1272-9-H50

**Chemicals, peptides, and recombinant proteins**		

Oxytocin	KKL Med	Cat#KM17618
Clozapine N-oxide	Enzo	Cat#BML-NS105-0025
Colchicine	MCE	Cat#HY-16569
DAPI	Sigma	Cat#D9542

**Experimental models: Organisms/strains**		

C57BL/6J mice	Charles River Laboratories	–
Ai14 mice	Jackson Laboratory	RRID:IMSR_JAX:007905
CRH-Cre mice	Jackson Laboratory	RRID:IMSR_JAX:011087

**Software and algorithms**		

ANY-maze	Stoelting	https://www.anymaze.com/support/downloads/
Zen Imaging Software	Zeiss	https://www.zeiss.com/corporate/en_de/global/home.html
Clampfit 10.0	Molecular Devices	–
GraphPad Prism 8.0	GraphPad Software	https://www.graphpad.com/scientific-software/prism/
ImageJ	NIH	https://imagej.nih.gov/ij/

**Other**		

Confocal microscope	Zeiss	LSM 800
Slide scanner	Olympus	VS120
Vibratome	Leica	VT-1000S
Digidata 1440 A	Molecular Devices	–
Multiclamp 700B amplifier	Molecular Devices	–

### Experimental model and study participant details

#### Animals

Male and female C57BL/6J mice older than 8 weeks were procured from Charles River Laboratories in Beijing and Hangzhou. The F1 offspring mice were generated using the purchased male and female mice, with the male offspring being used for all subsequent experiments. The corticotrophin-releasing hormone (CRH)-Cre (011087, B6;FVB-Tg(Crh-cre)1Kres/J, Jackson Laboratory), Ai14 (007908, B6;129S6-Gt(ROSA)26Sortm14(CAG-tdTomato)Hze/J, Jackson Laboratory) mice were used in the current study. Homozygous CRH-Cre mice were crossed with Ai14 mice to generate F1 generation of CRH:Ai14 mice with the male offspring being used for all subsequent experiments. Mice were provided *ad libitum* access to standard chow and water with a 12-hour light/dark cycle under controlled conditions (20-22°C, 30-70% humidity). All procedures were approved by the Shenzhen Institute of Advanced Technology (SIAT), Chinese Academy of Sciences (CAS) (approval number: SIAT-IACUC-250704-NS-XYL-A2993).

### Method details

#### Early life stress

The procedures were performed as previously described.^33^ After birth, each dam was assigned to care six pups, pups of either sex remained untouched until postnatal day 2 (PND2). Pups were randomly divided in 2 groups. The early life stress (ELS) group consisted of pups removed from their litter and isolated in small compartments for 4 h per day from PND2 to PND6 and for 8 h per day from PND7 to PND16 followed by being weaned at PND17. During the separation, we conducted ELS procedures during the light phase of the light/dark cycle (from 8:00 AM to 8:00 PM). Animals were maintained in heating plate was provided, maintaining constant temperature and humidity. The control group was not being separated and weaned until PND21 except. During cage changing, some old padding was transferred into another cage in order to attenuate stress. Additionally, during ELS stage, pups received intraperitoneal injections of OXT (1 mg/kg)^26^ once per day from PND2 to PND21.

#### Open field test

On postnatal day 56, an open field test^34^ was conducted to assess anxiety-like behavior in the mice. The open field apparatus consisted of a white-painted plywood box (40 × 40 × 40 cm), which was virtually divided into 25 equal-sized squares. The central 9 squares were defined as the center zone. Each mouse was individually placed in the center of the arena and allowed to explore freely for 5 minutes, the time spent in the center zone and the number of entries into the center zone was measured using ANY-maze Video Tracking Software. After each trial, the apparatus was thoroughly cleaned with 75% ethanol to eliminate odor cues from the previous subject. The time spent in the center zone and the number of entries into the center zone were used as indicators of anxiety-like behavior. We conducted the behavioral testing during the light phase of the light/dark cycle (from 1:00 PM to 7:00 PM).

#### Light/dark box test

A light-dark box test (LDT)^35^ at postnatal day 56 of the mice was performed to assess anxiety-like behavior. The LDT apparatus consisted of a black opaque acrylic chamber (42 cm × 21 cm × 25 cm) comprising light and dark in a 2:1 ratio (light compartment vs. dark compartment). Chambers were interconnected via a transitional aperture (height × width: 3 × 5 cm). Behavioral tracking was achieved through a digital video tracking system positioned vertically above the apparatus. Illumination intensities were maintained at 200 lux (light compartment) and 2 lux (dark compartment), verified using a calibrated lux meter (accuracy ± 10 Lux). Mice were habituated to the testing environment for 30 min before assessment. Each mouse was introduced to the light compartment, followed by being placed in the light compartment and recorded using digital video. Mice were allowed to freely explore the light and dark compartments for 15 minutes. The chamber was sterilized with 75% ethanol between trials to prevent olfactory confounders. The percentage of time spent in the light compartment, the number of entries into the light compartment, and the total distance traveled in the light compartment were analyzed and quantified to evaluate anxiety-like behavior using ANY-maze Video Tracking Software. We conducted the behavioral testing during the light phase of the light/dark cycle (from 1:00 PM to 7:00 PM).

#### DREADD manipulation

For experiments involved in chemogenetic activation of PVN^CRH^ neurons, CRH-Cre male mice received bilateral PVN microinjections of AAV9-EF1α-DIO-hM3D(Gq)-mCherry bilaterally at PND 28. Following 3 weeks, anxiety-like behavior and c-Fos staining were conducted. 30 min after a CNO (0.5 mg/kg, i.p.) or equal amounts of saline (i.p.) injection, the behaviors were recorded. In the chemogenetic inhibition experiments, CRH-Cre male mice received bilateral PVN microinjections of AAV9-EF1α-DIO-hM4D(Gi)-mCherry, the mice were subjected to behaviors, electrophysiological recording and c-Fos staining. The behaviors were recorded and analyzed 30 min after a 0.1 ml saline (i.p.) or CNO (5 mg/kg, i.p.) injection. Viral injection sites and expression were histologically validated post-experiment.

#### Immunofluorescence

Mice underwent terminal anesthesia (ketamine 150 mg/kg, xylazine 20 mg/kg, i.p.) followed by transcardial perfusion with PBS and 4% paraformaldehyde (PFA) in phosphate buffer. Brains were dissected and post-fixed in 4% PFA overnight (4°C), then cryoprotected in graded sucrose solutions (15% and 30%) until tissue sinking. Coronal sections (40 μm) were obtained using a cryostat (CM1950, Leica, Germany). Sections underwent three PBS washes (5 min each) prior to 2 h blocking at room temperature in 5% normal goat serum/1% Triton X-100. Sections were incubated in primary antibodies diluted in blocking solution (5% normal goat serum containing 1% Triton X-100 in PBS) at 4°C overnight. Primary antibodies: Rabbit anti-c-Fos (1:1000, 2250S, Cell Signaling), Rabbit anti-CRH (1:1000, T-4037, Peninsula Laboratories International) or Chicken anti-c-Fos (1:1000, 226009, Synaptic Systems). After PBS washes, sections were exposed to fluorescent-conjugated secondary antibodies for 2 h at room temperature protected from light. Cell nuclei were stained using 6-diamidino-2-phenylindole (DAPI, D9542, Sigma). Afterwards, these sections were mounted on slides, and coverslipped. Images were captured using Zeiss LSM 800 confocal and Olympus VS120 virtual microscopy systems. The secondary antibodies used were Alexa Fluor 488 Goat anti-Rabbit (1:1000, 111-547-003, Jackson Immuno Research), Alexa Fluor Cy5 Goat anti-Rabbit (1:1000, A21070, Invitrogen), and Alexa Fluor 488 Goat anti-Chicken (1:1000, A11039, Invitrogen) and Alexa Fluor 647 Goat anti Chicken (1:1000, A21449, Invitrogen). We analyzed one side of the paraventricular nucleus (PVN). The immunoreactive positive cells were manually quantified using ImageJ software.

#### Stereotaxic surgery

Mice at PND28, were anesthetized via intraperitoneal injection of ketamine/xylazine (150 mg/kg and 20 mg/kg body weight respectively). Cranial fur was depilated with electric clippers, then mice were fixed in a stereotaxic apparatus. The scalp was disinfected with 75% ethanol followed by povidone-iodine. Sagittal scalp incision (1.5 cm anterior and 0.5 cm posterior to the interaural line) was made to expose the skull. Virus or colchicine was uploaded into the microsyringe (bubble-free). PVN coordinates were localized according to mouse brain atlas. 200 nl viral solution or compound was stereotactically delivered to bilateral PVN at 1 nl/s. Microsyringe held for additional 10 min to minimize backflow. Subsequent procedures were performed as experimentally required.

#### Electrophysiological recordings

Following isoflurane anesthesia on PND57, rapid transcranial perfusion was performed using ice-cold choline-based solution under.^36^ 300 μm coronal sections containing PVN were sectioned using a precision vibrotome (VT-1000S, Leica) in an ice-cold choline-based solution. PVN-containing slices were transferred to 32°C oxygenated artificial cerebrospinal fluid for at least 30 min prior to recording. Whole-cell voltage clamp recording was performed using Multiclamp 700B amplifier and a Digidata 1440 A (Molecular Devices). Postsynaptic currents were recorded with a Cs-based internal solution. For targeted recording of sEPSCs and sIPSCs from CRH^+^ neurons using CRH:AI14 and CRH-Cre mice with virus injection in PVN. The mice received systemic OXT administration (1 mg/kg, i.p.) prior to the behaviors and electrophysiological recordings. The firing recording were performed with a K-based internal solution. The intrinsic excitability of PVN^CRH^ neurons was examined using CRH:Ai14 mice. The mice received OXT administration before behaviors and electrophysiological recordings. CRH^+^ neurons were injected with steady depolarizing currents (1 s, 0–30 pA, 5 pA steps). Electrode resistance was maintained at 4-6 MΩ throughout recordings. Data analysis utilized Clampfit 10.7 (Molecular Devices).

#### Statistical analysis

Statistical analysis in this study was performed using GraphPad Prism software. The following statistical methods were employed: one-way ANOVA, two-way ANOVA, t-test, and non-parametric tests. All data are presented as mean ± SEM. Statistical significance was denoted as ∗*P* < 0.05, ∗∗*P* < 0.01, and ∗∗∗*P*< 0.001.

## References

[1] Birnie M.T., Baram T.Z. (2025). The evolving neurobiology of early-life stress. Neuron.

[2] Shin S., Pribiag H., Lilascharoen V., Knowland D., Wang X.Y., Lim B.K. (2018). Drd3 Signaling in the Lateral Septum Mediates Early Life Stress-Induced Social Dysfunction. Neuron.

[3] Lupien S.J., McEwen B.S., Gunnar M.R., Heim C. (2009). Effects of stress throughout the lifespan on the brain, behaviour and cognition. Nat. Rev. Neurosci..

[4] Nemeroff C.B. (2016). Paradise Lost: The Neurobiological and Clinical Consequences of Child Abuse and Neglect. Neuron.

[5] LeMoult J., Humphreys K.L., Tracy A., Hoffmeister J.A., Ip E., Gotlib I.H. (2020). Meta-analysis: Exposure to Early Life Stress and Risk for Depression in Childhood and Adolescence. J. Am. Acad. Child Adolesc. Psychiatry.

[6] Ladd C.O., Owens M.J., Nemeroff C.B. (1996). Persistent changes in corticotropin-releasing factor neuronal systems induced by maternal deprivation. Endocrinology.

[7] Accarie A., Vanuytsel T. (2020). Animal Models for Functional Gastrointestinal Disorders. Front. Psychiatry.

[8] Vannucchi M.G., Evangelista S. (2018). Experimental Models of Irritable Bowel Syndrome and the Role of the Enteric Neurotransmission. J. Clin. Med..

[9] Wade M., Wright L., Finegold K.E. (2022). The effects of early life adversity on children's mental health and cognitive functioning. Transl. Psychiatry.

[10] Füzesi T., Daviu N., Wamsteeker Cusulin J.I., Bonin R.P., Bains J.S. (2016). Hypothalamic CRH neurons orchestrate complex behaviours after stress. Nat. Commun..

[11] McCall J.G., Al-Hasani R., Siuda E.R., Hong D.Y., Norris A.J., Ford C.P., Bruchas M.R. (2015). CRH Engagement of the Locus Coeruleus Noradrenergic System Mediates Stress-Induced Anxiety. Neuron.

[12] van Bodegom M., Homberg J.R., Henckens M.J.A.G. (2017). Modulation of the Hypothalamic-Pituitary-Adrenal Axis by Early Life Stress Exposure. Front. Cell. Neurosci..

[13] Lai M.C., Huang L.T. (2011). Effects of early life stress on neuroendocrine and neurobehavior: mechanisms and implications. Pediatr. Neonatol..

[14] Chen Y., Baram T.Z. (2016). Toward Understanding How Early-Life Stress Reprograms Cognitive and Emotional Brain Networks. Neuropsychopharmacology.

[15] Juruena M.F., Eror F., Cleare A.J., Young A.H. (2020). The Role of Early Life Stress in HPA Axis and Anxiety. Adv. Exp. Med. Biol..

[16] Yuan Y., Wu W., Chen M., Cai F., Fan C., Shen W., Sun W., Hu J. (2019). Reward Inhibits Paraventricular CRH Neurons to Relieve Stress. Curr. Biol..

[17] Qian W., Zhu T., Tang B., Yu S., Hu H., Sun W., Pan R., Wang J., Wang D., Yang L. (2014). Decreased circulating levels of oxytocin in obesity and newly diagnosed type 2 diabetic patients. J. Clin. Endocrinol. Metab..

[18] Neumann I.D., Slattery D.A. (2016). Oxytocin in General Anxiety and Social Fear: A Translational Approach. Biol. Psychiatry.

[19] Wan B., Zhang L., Wang X., Zhang R., Li L., Zhang Y., Chen Z., Hu C. (2025). Fam172a Mediates the Stimulation of Hypothalamic Oxytocin Neurons to Suppress Obesity-Induced Anxiety. Adv. Sci..

[20] Amico J.A., Mantella R.C., Vollmer R.R., Li X. (2004). Anxiety and stress responses in female oxytocin deficient mice. J. Neuroendocrinol..

[21] Nisbett K.E., Gonzalez L.A., Teruel M., Carter C.S., Vendruscolo L.F., Ragozzino M.E., Koob G.F. (2023). Sex and hormonal status influence the anxiolytic-like effect of oxytocin in mice. Neurobiol. Stress.

[22] Zhan S., Qi Z., Cai F., Gao Z., Xie J., Hu J. (2024). Oxytocin neurons mediate stress-induced social memory impairment. Curr. Biol..

[23] Nicolaides N.C., Kanaka-Gantenbein C., Pervanidou P. (2024). Developmental Neuroendocrinology of Early-Life Stress: Impact on Child Development and Behavior. Curr. Neuropharmacol..

[24] Shi D.D., Zhang Y.D., Ren Y.Y., Peng S.Y., Yuan T.F., Wang Z. (2021). Predictable maternal separation confers adult stress resilience via the medial prefrontal cortex oxytocin signaling pathway in rats. Mol. Psychiatry.

[25] Kikusui T., Takeuchi Y., Mori Y. (2004). Early weaning induces anxiety and aggression in adult mice. Physiol. Behav..

[26] Baracz S.J., Robinson K.J., Wright A.L., Turner A.J., McGregor I.S., Cornish J.L., Everett N.A. (2022). Oxytocin as an adolescent treatment for methamphetamine addiction after early life stress in male and female rats. Neuropsychopharmacology.

[27] Heim C., Young L.J., Newport D.J., Mletzko T., Miller A.H., Nemeroff C.B. (2009). Lower CSF oxytocin concentrations in women with a history of childhood abuse. Mol. Psychiatry.

[28] Meinlschmidt G., Heim C. (2007). Sensitivity to intranasal oxytocin in adult men with early parental separation. Biol. Psychiatry.

[29] Jurek B., Slattery D.A., Hiraoka Y., Liu Y., Nishimori K., Aguilera G., Neumann I.D., van den Burg E.H. (2015). Oxytocin Regulates Stress-Induced Crf Gene Transcription through CREB-Regulated Transcription Coactivator 3. J. Neurosci..

[30] Higashida H., Lopatina O., Yoshihara T., Pichugina Y.A., Soumarokov A.A., Munesue T., Minabe Y., Kikuchi M., Ono Y., Korshunova N., Salmina A.B. (2010). Oxytocin signal and social behaviour: comparison among adult and infant oxytocin, oxytocin receptor and CD38 gene knockout mice. J. Neuroendocrinol..

[31] Pati D., Harden S.W., Sheng W., Kelly K.B., de Kloet A.D., Krause E.G., Frazier C.J. (2020). Endogenous oxytocin inhibits hypothalamic corticotrophin-releasing hormone neurones following acute hypernatraemia. J. Neuroendocrinol..

[32] Zhang S., Zhang Y.D., Shi D.D., Wang Z. (2023). Therapeutic uses of oxytocin in stress-related neuropsychiatric disorders. Cell Biosci..

[33] Martín-Sánchez A., García-Baos A., Castro-Zavala A., Alegre-Zurano L., Valverde O. (2021). Early-life stress exacerbates the effects of WIN55,212-2 and modulates the cannabinoid receptor type 1 expression. Neuropharmacology.

[34] Xu Y., Jiang C., Wu J., Liu P., Deng X., Zhang Y., Peng B., Zhu Y. (2022). Ketogenic diet ameliorates cognitive impairment and neuroinflammation in a mouse model of Alzheimer's disease. CNS Neurosci. Ther..

[35] Linders L.E., Patrikiou L., Soiza-Reilly M., Schut E.H.S., van Schaffelaar B.F., Böger L., Wolterink-Donselaar I.G., Luijendijk M.C.M., Adan R.A.H., Meye F.J. (2022). Stress-driven potentiation of lateral hypothalamic synapses onto ventral tegmental area dopamine neurons causes increased consumption of palatable food. Nat. Commun..

[36] Keyes P.C., Adams E.L., Chen Z., Bi L., Nachtrab G., Wang V.J., Tessier-Lavigne M., Zhu Y., Chen X. (2022). Orchestrating Opiate-Associated Memories in Thalamic Circuits. Neuron.

